# Potential lipid-lowering effects of *Ulmus macrocarpa* Hance extract in adults with untreated high low-density lipoprotein cholesterol concentrations: A randomized double-blind placebo-controlled trial

**DOI:** 10.3389/fmed.2022.1000428

**Published:** 2022-11-01

**Authors:** Ye Li Lee, Sang Yeoup Lee

**Affiliations:** ^1^Integrated Research Institute for Natural Ingredients and Functional Foods, Yangsan, South Korea; ^2^Family Medicine Clinic and Biomedical Research Institute, Pusan National University Yangsan Hospital, Yangsan, South Korea; ^3^Department of Medical Education, Pusan National University School of Medicine, Yangsan, South Korea

**Keywords:** dietary supplements, dyslipidemia, *Ulmus macrocarpa* Hance, lipids, lipoprotein, randomized controlled trial

## Abstract

**Introduction:**

*Ulmus macrocarpa* Hance extract (UME) has demonstrated an antilipidemic effect *via* upregulation of the adenosine monophosphate-activated protein kinase pathway and regulation of lipid metabolism in both laboratory and animal studies. Therefore, we examined the effects and safety of UME on plasma lipids in adults with untreated high, low-density lipoprotein cholesterol (LDL-C) concentrations.

**Materials and methods:**

In the current double-blind placebo-controlled randomized clinical trial, 80 patients with untreated high LDL-C concentrations (130–190 mg/dl) were randomly allocated to either the “UME group” (received 500 mg UME as two capsules per day) or the “Placebo group” (received placebo containing cornstarch as two capsules per day) for 12 weeks. The primary outcome was the change in LDL-C concentration within the 12-week treatment period; secondary outcomes included changes in total cholesterol (TC), triglyceride, high-density lipoprotein cholesterol, apolipoprotein A1, and apolipoprotein B (ApoB) concentrations.

**Results:**

UME over 12 weeks led to a greater decrease in LDL-C, TC, and ApoB concentrations than did the placebo as follows: by 18.1 mg/dl (*P* < 0.001); 23.3 mg/dl (*P* < 0.001); 9.3 mg/dl (*P* = 0.018), respectively. When LDL-C, TC, and ApoB concentrations were expressed as a lsmeans percentage of the baseline concentration, they after 12 weeks of UME had greater % differences compared to the placebo as follows: by 11.9% (*P* < 0.001); 10.0% (*P* < 0.001); 8.6% (*P* < 0.05), respectively. However, no significant inter- and intra-group changes in liver enzyme, free fatty acid, anti-inflammatory marker, and fasting glucose concentrations were observed. None of the participants experienced notable adverse events.

**Discussion:**

UME causes a significant improvement in lipid profiles in adults with untreated high LDL-C concentrations.

**Clinical trial registration:**

[www.clinicaltrials.gov/], identifier [NCT03773315].

## Introduction

Dyslipidemia is recognized as one of the most common modifiable risk factors for developing atherosclerosis and subsequent ischemic heart disease (IHD) (1). The global burden of dyslipidemia has steadily increased over the past 30 years (2, 3). The World Health Organization (WHO) reported a global prevalence of hypercholesterolemia in adults aged ≥18 of 39% in 2008 (4). High LDL-cholesterol rapidly increased from the 15th leading risk for death in 1990 to the 8th in 2019 (2). The prevalence of dyslipidemia in the young population also is increasing (5). Compared to those without dyslipidemia, adults with dyslipidemia are at approximately twice the risk of developing cardiovascular disease (CVD), among the leading causes of mortality worldwide (1, 6). Further, hypercholesterolemia is estimated to cause 56% of IHDs and 18% of strokes worldwide (6).

The initial management of dyslipidemia involves optimizing lifestyle changes and correcting secondary exacerbating factors before beginning antilipemic drug use (7). Weight loss, changes in dietary macronutrient composition such as a Mediterranean-style diet, and physical activity, or the combination of them, contribute to triglyceride reduction (8). They remain important even when using medications (9). Lipid-lowering medications must also be administered to patients with higher CVD risk who do not respond to non-pharmacological therapy. Currently, statins are the most used therapeutic option for treating dyslipidemia as they reduce the risk of cardio-cerebrovascular events and mortality (10). Previous studies have reported on various statin drugs, such as lovastatin, simvastatin, atorvastatin, and rosuvastatin, which induce hypolipidemia *via* inhibiting β-hydroxy β-methylglutaryl-CoA reductase (HMGCR), a rate-limiting enzyme of the cholesterol biosynthetic pathway (11). Although statins are effective for lowering cholesterol and protecting against cardiovascular and cerebrovascular events, they may elicit side effects in some patients, including muscle- and skeletal-related adverse events (AEs) (pain, weakness, myopathy, and rhabdomyolysis), liver damage, increased risk of developing type 2 diabetes, memory loss, and confusion (12).

A focus-group study in Germany revealed that people use herbal medicine primarily to treat mild to moderate illnesses for all age groups and prevent illnesses or promote health, especially for the elderly. Also, they were aware of the limits of herbal medicine for severe illnesses (13). Although these standard lipid-lowering treatments should be used in patients with high or very high CVD risk, functional foods may be recommended for individuals with borderline lipid profile levels or drug intolerance (14). In recent years, lipid-lowering nutraceuticals and functional foods identified through clinical studies have included phytosterols, oat β-glucan, chitosan, and probiotic lactobacillus as inhibitors of intestinal cholesterol absorption; monacolin K as an inhibitor of liver cholesterol synthesis; green tea catechin extract and milk polar lipids as inducers of low-density lipoprotein cholesterol (LDL-C) excretion; and spirulina supplementation, krill oil, turmeric, and curcuminoids as nutraceuticals with mixed mechanisms of action (14).

Recently, *Ulmus macrocarpa* Hance extract (UME) exhibited potential as supporting therapy for lowering plasma total cholesterol (TC), triglyceride (TG), and LDL-C concentrations in hypercholesterolemic conditions by regulating the adenosine monophosphate-activated protein kinase (AMPK) pathway and lipid metabolism *in vitro* and *in vivo* using oleic acid (OA)-treated HepG2 cells and high-cholesterol diet (HCD)-induced hyperlipidemia rats (15). However, no randomized, placebo-controlled trial in humans has explored the effects and safety of UME in hyperlipidemia. We hypothesized that UME has a lipid profile-improving effect in adults based on previous studies. Thus, this randomized, double-blinded, placebo-controlled trial aimed to investigate the impact of UME administration for 12 weeks on lipid profiles in adults with untreated high LDL-C concentrations and to test its safety.

## Materials and methods

### Study participants and ethical aspects

This study was approved by the Institutional Review Board at Pusan National University Yangsan Hospital (IRB 02-2018-029, 8 October 2018). It was conducted in accordance with the principles of the Declaration of Helsinki and the Korean Good Clinical Products guidelines. Written informed consent was obtained from all study participants recruited through advertisements at a tertiary hospital in Yangsan, South Korea. The trial was conducted between April 2019 and October 2019. The trial was registered in the Registry Clinical Trial.^1^

According to the clinical practice guideline of the Korean Society of Lipid and Atherosclerosis for the Korean population, statins are recommended for patients with LDL-C concentration ≥190 mg/dl, irrespective of the level of risk. Also, statins are considered when LDL-C concentration ≥130 mg/dl persists even after weeks or months of lifestyle modification for moderate-risk and low-risk groups (16). Therefore, participants ≥20 years of age and with LDL-C concentrations ranging from 130 to 190 mg/dl were eligible for the study. Participants using of lipid-lowering drugs within the previous 3 months; with a history of cerebrovascular diseases (such as cerebral infarction, cerebral hemorrhage, etc.) or heart disease (such as unstable angina, myocardial infarction, heart failure, etc.) for which lesser than 6 months had passed since hospital discharge; with abnormal liver or renal function (aspartate aminotransferase or alanine aminotransferase concentration more than two times the upper limit of normal; creatinine concentration more than two times the upper limit of normal; or proteinuria, defined as a urinalysis dipstick reading of ≥2+); with hyperthyroidism or hypothyroidism; with diabetes (diagnosed clinically or with a fasting glucose concentration >126 mg/dl); with uncontrolled hypertension [blood pressure (BP) ≥160/100 mmHg); with any cancer; with use of any medication or supplements within the preceding 1 month, which could have caused a change in body weight, including anti-absorptive agents, appetite suppressors, and any other hormonal products; with psychiatric disorder; alcohol abuser; who had quit smoking within 3 months of enrollment; with severe gastrointestinal symptoms; or any allergic reaction to the involved ingredients; or pregnant or lactating women were excluded.

### Study design

The study was a randomized, placebo-controlled, double-blinded controlled trial. Simple randomization of the two study groups was performed using a random number table. The table of random numbers was generated using the Excel^®^ random number macro (Microsoft Corp., Redmond, WA, USA). Participants were assigned sequentially randomized numbers, and these randomization codes were held by the company that manufactured the UME and the dummy placebo (Supplementary Table 1). The authors who selected the study participants and those who performed the measurements were blinded to the randomization assignments.

After the baseline assessment, participants were randomly allocated to either the UME-supplemented group or the placebo-supplemented group. Participants were requested to log when they took the supplement in a diary, which was turned in along with the bottle to the researcher at every visit. Compliance was assessed by pill counting of the supplements that participants brought with them at each visit; if more than 20% were unused, the participant was considered to have dropped out of the study. Adherence rates of ≥80% were required for optimal therapeutic efficacy. This cut-off is widely used as a conventional threshold for good adherence (17). Each participant was instructed to visit the clinic at 6 weeks (±7 days) and 12 weeks (±7 days) after the initiation of treatment. BP and blood tests, including the lipid profile, were performed at each visit. BP was measured three times in the sitting position after a 10-min rest using a model BP-203 RV II device (Colin Corp., Aichi, Japan), and the average was used. Physical activity and nutrition assessments were performed at baseline and 12 weeks (±7 days) after treatment. Participants were counseled to maintain their usual lifestyle and diet during the 12 weeks of the study.

### Intervention

Participants were randomly assigned to the UME group (supplied by Naturetech Co., Ltd., Seoul, South Korea) or the placebo group. The UME group was administered 500 mg UME/day orally, that is, one 250 mg capsule 30 min after breakfast and dinner, for 12 weeks. The UME contained a mean 5.08 mg of total catechin/g, obtained through hydrothermal extraction, as determined by HPLC analysis. The proportions of each catechin were (−)-epigallocatechin (EGC, 37.19%), (−)-epigallocatechin-gallate (EGCG, 3.58%), (−)-epicatechin (EC, 38.04%), and (−)-epicatechin-gallate (ECG, 21.19%). The placebo group was administered the same quantity of the placebo identically. The placebo was identical in appearance to the UME capsule but was filled with corn starch. Based on the results of a previous animal study, which showed that the efficacious dose of UME for lowering lipids was 100 mg/kg (15), the dose used in the animal subjects was converted to a human equivalent dose based on the person’s body surface area, that is, 480 mg for individuals weighing 60 kg. Thus, 500 mg/60 kg was selected as the final dose. In the preclinical toxicity test, this dose of UME satisfied all standards for hazardous substances such as heavy metals, microorganisms, safe pesticides, and residual sulfur dioxide. Furthermore, it reduced hepatotoxicity in experimental animals (15).

### Measurements of efficacy

The primary study outcome measure was the change in LDL-C concentration within the 12-week treatment period. Secondary outcome measures were changes in TC, TG, high-density lipoprotein cholesterol (HDL-C), apolipoprotein A1 (ApoA1), apolipoprotein B (ApoB), free fatty acid, and high-sensitivity C-reactive protein (hs-CRP) concentrations.

#### Biochemical measurements

All laboratory analyses were performed in a central laboratory. After a 12-h overnight fast, blood samples were collected at the baseline and at 6 and 12 weeks after the randomization to evaluate the antilipidemic effect of UME and monitor any potential adverse effects. Plasma hs-CRP was measured by latex particle-enhanced immunoturbidimetric assay on the AU5800 chemistry analyzer (Beckman Coulter, Brea, CA, USA). Free fatty acids were determined by an enzymatic colorimetric method assay (NEFA-HR2, ACS-ACOD; Wako Chemicals, Neuss, Germany) on the Cobas 8000 c502 analyzer (Roche Diagnostics, Mannheim, Germany). Plasma TC, TG, HDL-C, and LDL-C concentrations were measured using an enzymatic colorimetric assay on the AU5800 chemistry analyzer (Beckman Coulter, Brea, CA, USA). ApoA1 and ApoB concentrations were measured using an immunoturbidimetric method (Tina-quant, Roche Diagnostics, Mannheim, Germany) on the Cobas 8000 c502 analyzer (Roche Diagnostics, Mannheim, Germany). Serum liver enzyme, glucose, and creatinine concentrations were measured using the TBA200FR biochemical analyzer (Toshiba Co. Ltd., Tokyo, Japan).

#### Dietary intake and physical activities assessments

At the baseline and after 12 weeks of the trial, participants were asked to answer a questionnaire on dietary intake and physical activities that may influence changes in lipid profiles. Information on the nutritional intake of participants was collected using the 24-h dietary recall method. The CAN-Pro version 4.0 (Computer Aided Nutritional Analysis Program for Professionals 4.0; Korean Nutrition Society) was used for nutrient analysis of the surveyed dietary intake. The frequency, intensity, and type of physical activities performed by participants during the preceding 7 days were reported using the International Physical Activity Questionnaires (IPAQ) (18). The number of physical activities was represented as the metabolic equivalent of task (METs).

### Safety and tolerability assessments

All randomized participants exposed to at least one dose of the study intervention were included in the safety analysis. All randomized participants exposed to at least one dose of the study intervention were included in the safety analysis. Per protocol, safety was assessed at each study visit based on AEs, vital signs, physical examination, and laboratory test results (complete blood counts, liver enzymes, glucose, and creatinine). Reports of any other AEs or unpredicted allergic reactions were collected throughout the study. All AEs were coded using version 21.0 of the Medical Dictionary for Regulatory Activities.

### Statistical analyses

Data were presented as either mean ± SD, median [IQR], or mean (95% CI) for continuous variables and number (%) for categorical variables. We used MedCalc version 19.4.1 (MedCalc Software Ltd., Ostend, Belgium) to calculate the sample size based on a previous similar study (19). The estimated sample size was determined to be 32 subjects per group for 80% power to detect a difference of 14.4 mg/dl in the LDL-C concentrations, assuming an SD of 20.3 mg/dl in the primary outcome and an α error of 5% (19). By considering the changes in ox-LDL level as a main outcome, a 1/4 0.05, power of 80%, and anticipating a probable dropout rate of 20% during the intervention course, 40 patients were recruited in each group. Eighty participants (40 per group) were recruited, with an assumed dropout rate of 20%. Intention-to-treat (ITT) was the primary analysis for comparisons of outcomes between the UME and placebo groups, with multiple imputation of missing data (*n* = 80). Because the percentage of missing values at the 12-week follow-up was 11.3% for all variables, 5 imputed data sets were created, and the results of the analyses from the different imputed data sets were pooled according to Rubin’s rules using R software version 3.6.2 (R Foundation for Statistical Computing). Multivariate imputation by the chained equations algorithm was used with the predictive mean matching method. A per-protocol (PP) analysis was also performed (*n* = 71) to assess the effectiveness of the supplementation. Shapiro–Wilk’s test was used to test the normality assumption for all variables. Intergroup comparisons of baseline characteristics were performed using the two-sample *t*-test for continuous variables (or Mann–Whitney’s U test for non-parametric continuous variables) and the Chi-square test for categorical variables (or Fisher’s exact test for non-parametric categorical variables). ANCOVA or rank ANCOVA was used for the main analysis, with adjustment for each baseline variable and baseline dietary fat intake percentage as covariates. Model assumptions were checked by histograms, normal probability plots, and residual scatter plots. The change from baseline to week 12 in outcomes was expressed as a lsmean percentage of the baseline levels using an ANCOVA model. *P*-values < 0.05 were considered statistically significant. Data were analyzed using SPSS Statistics 25.0 software (IBM Corp., Armonk, NY, USA) and R software version 4.1.2.^2^

## Results

### Consolidated standards of reporting trials flow diagram and baseline characteristics of the subjects

**FIGURE 1:**
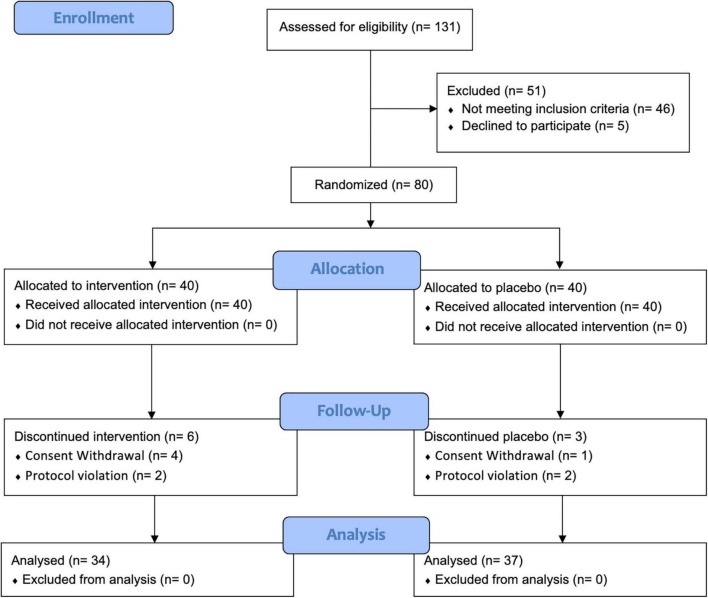
Consolidated standards of reporting trials (CONSORT) flow diagram.

The flow of participants through the controlled interventional trial is depicted in a consolidated standards of reporting trials (CONSORT) conform diagram (Figure 1). A total of 131 participants were screened. Of them, 80 (mean age 50.6 ± 9.8 years) were included in this study and randomly allocated to the UME or placebo group. The median LDL-C concentration was 147.0 mg/dl [interquartile range, IQR 137.0–162.5]. Four participants in the UME group and one in the placebo group withdrew from the study for personal reasons; this was not associated with any adverse effects. Two participants in each group were excluded due to protocol violations of non-compliance. Overall, 71 subjects (88.8%) completed the trial. Two (5%) subjects had comorbid disorders (one osteopenia and one irritable bowel syndrome) in the UME group, and three (7.5%) had comorbid disorders (one osteopenia and two hypertension) in the placebo group. Randomization was successful, as most variables were comparable between the two groups, and no significant differences were observed in the baseline demographic or anthropometric characteristics between the groups except daily fat intake (Table 1). There were no significant changes in the total calorie intake, macronutrient (carbohydrate, fat, and protein) intake, and physical activities checked at the baseline and 12 weeks of the trial among the participants, reflecting no additional effects that might have influenced the lipid profile, aside from the intervention (Table 2). During the entire study period, the double-blind requirement was maintained.

**TABLE 1:** Baseline characteristics of the study group.

Variables	Intention-to-treat population			Per protocol population		
	UME group (*n* = 40)	Placebo group (*n* = 40)	*P* ^1^	UME group (*n* = 34)	Placebo group (*n* = 37)	*P* ^1^
Age, year	50.6 ± 10.1	50.7 ± 9.6	0.955	49.2 ± 9.9	50.1 ± 9.4	0.685
Male, %	15 (37.5)	14 (35.0)	0.816	13 (38.2)	14 (37.8)	0.973
BMI, kg/m^2^	24.8 ± 2.9	24.8 ± 3.9	0.908	24.7 ± 2.8	25.0 ± 4.0	0.673
Systolic BP, mmHg	126.0 ± 13.9	127.2 ± 13.4	0.696	126.4 ± 14.1	128.3 ± 12.8	0.551
Diastolic BP, mmHg	82.6 ± 8.3	82.4 ± 10.7	0.935	82.4 ± 8.4	83.4 ± 10.2	0.676
Alcohol drinker, %	11 (27.5)	5 (12.5)	0.244	11 (32.3)	5 (13.5)	0.165
Moderate^2^	9 (22.5)	4 (10.8)		9 (26.5)	4 (10.8)	
Heavy^3^	2 (5.0)	1 (2.5)		2 (5.9)	1 (2.7)	
Current smoker, %	3 (7.5)	1 (2.5)	0.615	3 (8.8)	1 (2.7)	0.344
Energy intake, Kcal/day	1,754.4 ± 841.2	1,516.8 ± 396.9	0.110	1,812.5 ± 894.2	1,533.6 ± 404.5	0.090
Carbohydrate, %	58.7 ± 9.6	61.6 ± 9.6	0.173	57.9 ± 9.9	61.0 ± 9.2	0.166
Fat, %	25.4 ± 7.4	22.0 ± 7.4	0.048	25.9 ± 7.5	22.5 ± 6.6	0.044
Protein, %	15.8 ± 2.6	15.7 ± 3.3	0.807	15.8 ± 2.7	15.8 ± 3.3	0.954
IPAQ, METs	982.5 [535.5–2,283.5]	1,428.0 [714.0–2,171.0]	0.870	949.5 [495.0–2,274.0]	1,470.0 [724.5–2,245.5]	0.696
Adherence, %	93.7 ± 5.8	94.1 ± 4.4	0.718	94.1 ± 5.7	94.3 ± 4.1	0.889

Values are mean ± SD, median [IQR] or *n* (%). UME, *Ulmus macrocarpa* Hance extract; BMI, body mass index; BP, blood pressure; IPAQ, international physical activity questionnaires; MET, metabolic equivalent task.

^1^*P*-value by two-sample *t*-test for parametric variables, Mann–Whitney’s U test for non-parametric variables, and Chi-square test, or Fishers exact test for categorical variables.

^2^Moderate, 2 drinks or less in a day for men or 1 drink or less in a day for women, on days when alcohol is consumed.

^3^Heavy, more than moderate.

**TABLE 2:** Energy intake and physical activity between the two groups for 12 weeks.

	UME group		Placebo group		Adjusted difference of UME vs. placebo over 12 weeks	*P* ^1^
	Baseline	12 weeks	Baseline	12 weeks		
**Intention to treat (*n* = 80)**						
Energy intake, Kcal/day	1,754.4 ± 841.2	1,769.0 ± 601.2	1,516.8 ± 396.9	1,533.8 ± 467.2	135.02 (−75.20, 345.24)	0.205
Carbohydrate, %	58.7 ± 9.6	57.2 ± 10.9	61.6 ± 9.6	59.1 ± 12.9	−1.49 (−6.89, 3.91)	0.584
Fat, %	25.4 ± 7.4	25.9 ± 9.0	22.0 ± 7.4	24.4 ± 9.9	0.73 (−3.55, 5.01)	0.734
Protein, %	15.8 ± 2.6	16.0 ± 3.4	15.7 ± 3.3	17.4 ± 6.1	−1.49 (−3.63, 0.65)	0.169
IPAQ, METs	982.5 [515.3–2,288.3]	1,282.5 [459.0–1,750.1]	1,428.0 [703.5–2,182.5]	1,490.0 [681.8–2,398.0]	3.44 (−570.47, 577.35)	0.445
**Per protocol (*n* = 71)**						
Energy intake, Kcal/day	1,812.5 ± 894.2	1,783.1 ± 638.4	1,533.6 ± 404.5	1,563.7 ± 472.5	104.07 (−130.52, 338.67)	0.379
Carbohydrate, %	57.9 ± 9.9	57.1 ± 11.5	61.0 ± 9.2	58.3 ± 12.9	−1.00 (−6.95, 4.94)	0.738
Fat, %	25.9 ± 7.5	25.7 ± 9.6	22.5 ± 6.6	24.9 ± 10.0	0.15 (−4.62, 4.93)	0.949
Protein, %	15.8 ± 2.7	16.1 ± 3.4	15.8 ± 3.3	17.7 ± 6.2	−1.68 (−4.04, 0.67)	0.159
IPAQ, METs	949.5 [495.0–2,278.8]	1,282.5 [476.5–1,686.3]	1,470.0 [714.0–2,297.0]	1,584.0 [685.5–2,433.0]	−22.21 (−656.14, 611.73)	0.488

Values are mean ± SD or median [IQR] or mean (95% CI). UME, *Ulmus macrocarpa* Hance extract; IPAQ, international physical activity questionnaires; MET, metabolic equivalent task.

^1^ANCOVA or rank ANCOVA adjusted for each baseline value as covariates over the 12-week period.

### Primary outcome

**FIGURE 2:**
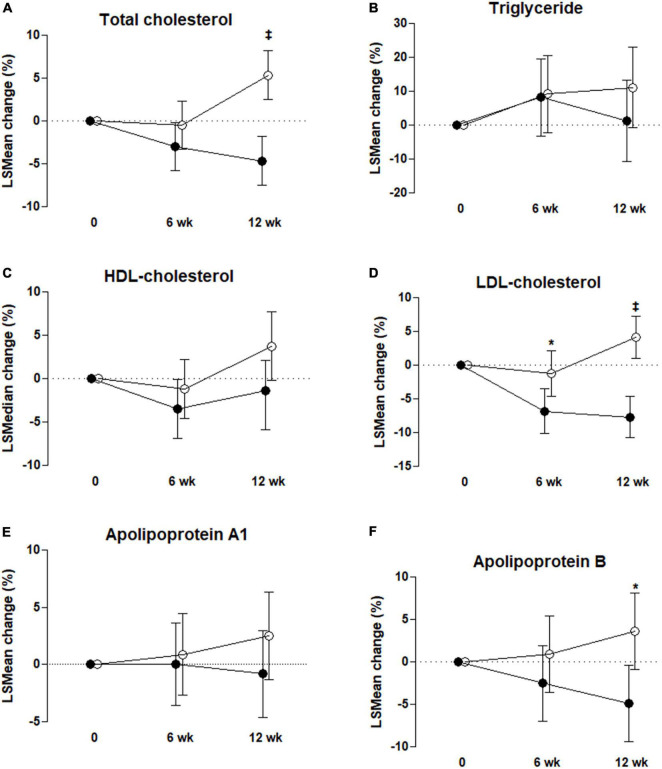
Percentage change from baseline to 6 and 12 weeks for total cholesterol **(A)**, triglyceride **(B)**, high-density lipoprotein (HDL)-cholesterol **(C)**, low-density lipoprotein (LDL)-cholesterol **(D)**, apolipoprotein A1 **(E)**, and apolipoprotein B **(F)** in the control group, °, and UME group, ∙. Values are mean ± SD except for triglyceride and HDL-cholesterol (median with IQR). *P < 0.05, ^‡^*P* < 0.001, *P*-value by ANCOVA or rank ANCOVA with adjustment for each baseline value and baseline dietary fat intake % as covariates; intent-to-treat analysis.

Table 3 shows that the LDL-C concentration of the UME group was significantly lower than in the placebo group after 6 and 12 weeks. In the ITT analysis, the concentrations of LDL-C were significantly decreased in the UME group compared to those in the placebo group at 6 weeks, by 8.02 mg/dl (95% CI: −15.37, −0.67; *P* = 0.033), and 12 weeks, by 18.05 mg/dl (95% CI: −25.00, −11.10; *P* < 0.001). The PP analysis also revealed that the LDL-C concentration in the UME group had decreased by 8.85 mg/dl (95% CI: −16.55, −1.15; *P* = 0.025) and 20.28 mg/dl (95% CI: −27.50, −13.07; *P* < 0.001) after 6 and 12 weeks of treatment, respectively, compared with that in the placebo group (Table 4). When LDL-C concentration was expressed as a lsmean percentage of the baseline concentration, LDL-C concentration of the UME group after 12 weeks demonstrated an 11.86% decrease compared to the placebo group. This intergroup difference in LDL-C concentrations was significant at the last visit, with an overall percentage change of −7.69 vs. 4.17% in the UME and placebo groups, respectively, from baseline (Figure 2, *P* < 0.001).

**TABLE 3:** Primary and secondary outcome measures of the two groups (intention-to-treat population).

	UME group (*n* = 40)			Placebo group (*n* = 40)			Adjusted difference of UME vs. placebo			
	Baseline	6 weeks	12 weeks	Baseline	6 weeks	12 weeks	Δ 6 weeks	*P* ^1^	Δ 12 weeks	*P* ^1^
LDL-C, mg/dl	152.5 ± 19.5	144.1 ± 21.0	139.0 ± 18.8	147.7 ± 17.1	145.6 ± 17.9	154.4 ± 19.9	−8.02 (−15.37, −0.67)	0.033	−18.05 (−25.00, −11.10)	<0.001
TC, mg/dl	238.2 ± 27.5	230.1 ± 30.7	225.3 ± 26.0	233.7 ± 27.0	231.7 ± 23.4	245.6 ± 28.0	−5.07 (−14.81, 4.67)	0.303	−23.29 (−33.64, −12.94)	<0.001
TG, mg/dl	104.5 [83.0–149.5]	123.0 [77.8–187.3]	104.0 [83.2–157.8]	120.0 [77.3–169.5]	119.0 [86.0–156.3]	117.0 [91.0–159.0]	13.38 (−10.13, 36.89)	0.423	10.42 (−11.53, 32.36)	0.657
HDL-C, mg/dl	58.4 ± 10.6	56.2 ± 12.7	56.7 ± 11.5	56.9 ± 14.3	55.9 ± 13.7	58.8 ± 13.9	−1.01 (−3.96, 1.94)	0.498	−3.23 (−6.64, 0.18)	0.063
ApoA1, mg/dl	148.0 ± 19.1	147.8 ± 23.6	146.0 ± 20.9	144.3 ± 26.5	144.8 ± 28.3	146.4 ± 25.1	−0.95 (−8.27, 6.37)	0.797	−3.61 (−11.16, 3.95)	0.345
ApoB, mg/dl	119.7 ± 15.9	116.8 ± 21.3	114.1 ± 20.9	123.8 ± 20.3	122.9 ± 18.6	126.7 ± 22.6	−3.69 (−11.21, 3.82)	0.331	−9.31 (−16.95, −1.66)	0.018
FFA, mg/dl	403.0 [277.3–551.0]		419.0 [242.0–615.0]	413.0 [312.0–551.8]		356.5 [259.5–520.8]	–	–	129.61 (−10.55, 248.67)	0.100
hs-CRP, mg/dl	0.1 [0.0–0.1]		0.1 [0.0–0.1]	0.1 [0.0–0.2]		0.1 [0.0–0.1]	–	–	−0.09 (−0.03, 0.20)	0.043

Values are mean ± SD, median [IQR] or mean (95% CI). UME, *Ulmus macrocarpa* Hance extract; LDL-C, low-density lipoprotein cholesterol; TC, total cholesterol; TG, triglyceride; HDL-C, high-density lipoprotein cholesterol; ApoA1, apolipoprotein A1; ApoB, apolipoprotein B; FFA, free fatty acid; hs-CRP, high-sensitivity C-reactive protein. FFA and hs-CRP, not measured at 6 weeks.

^1^ANCOVA or rank ANCOVA adjusted for each baseline value and baseline dietary fat intake% as covariates over the 12-week period.

**TABLE 4:** Primary and secondary outcome measures of the two groups (per protocol population).

	UME group (*n* = 34)			Placebo group (*n* = 37)			Adjusted difference of UME vs. placebo			
	Baseline	6 weeks	12 weeks	Baseline	6 weeks	12 weeks	Δ 6 weeks	*P* ^1^	Δ 12 weeks	*P* ^1^
LDL-C, mg/dl	153.2 ± 20.8	142.5 ± 22.1	138.5 ± 19.3	147.1 ± 16.8	146.7 ± 17.7	155.3 ± 20.4	–8.85 (–16.55, –1.15)	0.025	–20.28 (–27.50, –13.07)	<0.001
TC, mg/dl	238.0 ± 28.9	230.0 ± 32.4	223.5 ± 26.4	233.0 ± 25.9	232.5 ± 23.4	247.5 ± 28.2	–6.50 (–16.80, 3.80)	0.212	–27.18 (–37.66, –16.69)	<0.001
TG, mg/dl	101.0 [81.8–142.8]	126.5 [79.3–181.8]	105.0 [87.0–157.3]	134.0 [78.5–174.0]	121.0 [88.0–162.5]	117.0 [94.0–162.5]	–11.94 (–10.05, 33.92)	0.517	3.55 (–14.35, 21.45)	0.991
HDL-C, mg/dl	58.7 ± 9.7	56.3 ± 12.6	56.4 ± 11.7	56.7 ± 14.4	55.8 ± 13.9	58.9 ± 14.4	–1.48 (–4.51, 1.54)	0.331	–3.43 (–7.29, –0.24)	0.037
ApoA1, mg/dl	148.2 ± 18.6	147.1 ± 22.0	145.8 ± 20.9	145.1 ± 26.1	145.1 ± 28.6	147.2 ± 25.5	–1.59 (–9.05, 5.88)	0.673	–3.43 (–10.92, 4.05)	0.363
ApoB, mg/dl	119.7 ± 16.4	116.2 ± 22.7	113.3 ± 21.3	123.6 ± 20.7	123.9 ± 18.2	127.9 ± 22.9	–5.76 (–13.83, 2.31)	0.159	–11.51 (–19.57, –3.46)	0.006
FFA, mg/dl	389.5 [293.8–545.0]		427.5 [285.5–669.5]	414.0 [307.0–551.5]		362.0 [261.0–554.0]	–	–	100.68 (–15.20, 216.56)	0.253
hs-CRP, mg/dl	0.1 [0.0–0.1]		0.1 [0.0–0.1]	0.1 [0.0–0.2]		0.1 [0.0–0.1]	–	–	0.07 (–0.03, 0.17)	0.064

Values are mean ± SD, median [IQR] or mean (95% CI). UME, *Ulmus macrocarpa* Hance extract; LDL-C, low-density lipoprotein cholesterol; TC, total cholesterol; TG, triglyceride; HDL-C, high-density lipoprotein cholesterol; ApoA1, apolipoprotein A1; ApoB, apolipoprotein B; FFA, free fatty acid; hs-CRP, high-sensitivity C-reactive protein. FFA and hs-CRP, not measured at 6 weeks.

^1^ANCOVA or rank ANCOVA adjusted for each baseline value and baseline dietary fat intake% as covariates over the 12-week period.

### Secondary outcome

As shown in Table 3, based on ITT analysis, the UME group presented significantly decreased TC and ApoB concentrations, which were reduced by 23.29 and 9.31 mg/dl, respectively, compared with placebo group after 12 weeks of treatment (95% CI: −33.64, −12.94; *P* < 0.001 and 95% CI: −16.95, −1.66; *P* = 0.018, respectively). Also, based on PP analysis (Table 4), in UME group, TC and ApoB concentrations were significantly lower (decreased by 27.18 and 11.51 mg/dl, respectively) than those of the placebo group after 12 weeks of treatment (95% CI: −37.66, −16.69 mg/dl; *P* < 0.001 and 95% CI: −19.57, −3.46 mg/dl; *P* = 0.006, respectively). Moreover, in the UME group, the HDL-C concentration was significantly reduced, by 3.43 mg/dl (95% CI: −7.29, −0.24; *P* = 0.037), compared with the placebo group after 12 weeks of treatment, but only in the PP analysis. When TC and ApoB concentrations were expressed as a lsmean percentage of the baseline concentrations, TC and ApoB concentrations after 12 weeks of UME supplementation demonstrated a 10.02% (*P* < 0.001) and 8.56% (*P* = 0.01) decrease, respectively, compared to placebo (Figure 2). However, secondary outcomes, including TG, HDL-C, ApoA, and free fatty acids concentrations, did not differ between the two groups throughout the study period.

### Safety

All subjects completed the protocol without any adverse or serious AEs. There were no subject complaints in either of the groups. After the 12-week trial, there were two cases of diastolic BP exceeding 100 mmHg in the placebo group but none in the UME group. No significant changes in liver enzymes, glucose, or creatinine concentrations were observed between the two groups during the 12-week trial. Comparatively, diastolic BP in the UME group was lower than in the placebo group after 12 weeks of the trial (mean difference: −3.44 mmHg, *P* = 0.006). However, there was no significant difference in the systolic BP (Table 5).

**TABLE 5:** Laboratory findings evaluating the adverse effects.

	UME group		Placebo group		Adjusted difference of UME vs. placebo over 12 weeks	*P* ^1^
	Baseline	12 weeks	Baseline	12 weeks		
**Intention-to-treat (*n* = 80)**						
Systolic BP, mmHg	126.0 ± 13.9	123.7 ± 14.3	127.2 ± 13.4	125.5 ± 12.1	–0.73 (–5.32, 3.86)	0.751
Diastolic BP, mmHg	82.6 ± 8.3	80.1 ± 11.2	82.4 ± 10.7	83.4 ± 9.9	–3.44 (–6.82, –0.06)	0.046
AST, IU/L	25.1 ± 6.9	24.1 ± 6.8	26.0 ± 7.2	26.7 ± 8.9	–2.28 (–5.71, 1.15)	0.190
ALT, IU/L	22.1 ± 11.2	23.2 ± 12.6	24.5 ± 13.3	26.0 ± 17.7	–1.49 (–7.76, 4.78)	0.637
Creatinine, mg/dl	0.75 ± 0.18	0.74 ± 0.18	0.73 ± 0.18	0.73 ± 0.20	–0.01 (–0.06, 0.04)	0.740
Glucose, mg/dl	94.6 ± 9.3	93.8 ± 8.1	93.4 ± 9.9	93.1 ± 11.6	0.18 (–3.82, 4.18)	0.929
**Per protocol (*n* = 71)**						
Systolic BP, mmHg	126.4 ± 14.1	122.9 ± 14.9	128.3 ± 12.8	125.2 ± 12.0	–1.00 (–5.90, 3.89)	0.684
Diastolic BP, mmHg	82.4 ± 8.4	79.7 ± 11.6	83.4 ± 10.2	83.8 ± 9.7	–3.33 (–6.96, –0.29)	0.071
AST, IU/L	25.7 ± 7.2	23.6 ± 6.5	25.4 ± 4.9	26.6 ± 9.1	3.20 (–0.40, 6.80)	0.081
ALT, IU/L	23.1 ± 11.7	22.5 ± 12.6	23.8 ± 10.8	26.1 ± 18.3	3.16 (–3.09, 9.42)	0.317
Creatinine, mg/dl	0.76 ± 0.18	0.75 ± 0.18	0.74 ± 0.18	0.74 ± 0.18	0.01 (–0.03, 0.04)	0.752
Glucose, mg/dl	95.0 ± 9.7	93.8 ± 8.2	93.4 ± 10.2	93.3 ± 11.9	0.24 (–4.15, 4.63)	0.913

Values are mean ± SD or mean (95% CI). UME, *Ulmus macrocarpa* Hance extract; BP, blood pressure; AST, aspartate transaminase; ALT, alanine transaminase.

^1^ANCOVA adjusted for each baseline value as covariates over the 12-week period.

## Discussion

*Ulmus macrocarpa* Hance (UMH) is a large shrub endemic to the Far East. The stem and root bark of UMH have been used as traditional herbs to treat various conditions such as swelling, stomach disease, enteritis, dysuria, skin disease, mastitis, and arthritis (15, 20). Catechins, such as EGC, EGCG, EC, and EC, have several health benefits, such as antioxidant, antimicrobial, anti-inflammatory, and antiviral activities (21–23). Although catechins are the main components of green tea and UMC, EGCG is the most abundant catechin in green tea, and EGC and EC are the most abundant catechin in UMC. Green tea extract could suppress the mRNA level of HMGCR and increase the level of LDL receptors, leading to a lowered cholesterol level in mice fed with high-fat and high-sucrose diets. EGCG and EC could lower TC, LDL-C, and TG and increase HDL-C in hyperlipidemic rats (21–23). Green tea, containing catechin, was shown to remarkably reduce concentrations of LDL-cholesterol in humans (21–23). Previous studies have reported that UME has significant pharmacological potential, including antimicrobial, antioxidative, antiallergic, anti-inflammatory, antiplatelet, antihypertensive, and vasorelaxant effects (15, 20, 24). Recent studies have demonstrated that UME attenuates testosterone propionate-induced benign prostate hyperplasia *via* its pro-apoptotic and anti-proliferative activities (25); inhibits *Heliobacter pylori* colonization synergistically, especially when used in combination with *Rubus crataegifolius* (26); and prevents anti-photoaging of the skin by activating antioxidant enzymes and inhibiting the mitogen-activated protein kinase pathways (27). However, no study has assessed the effects of UME on lipid profiles in humans. Cardiovascular disease is still the major cause of morbidity and mortality. Despite the availability of different pharmacological drugs, new approaches are needed due to side effects and the general skepticism of many patients. Therefore, this study was designed as a primary prevention approach.

Our study evaluated the positive effect of UME on lipid profiles, which is another potential use of UME. To the best of our knowledge, this is the first randomized, double-blind, placebo-controlled trial to investigate the efficacy and safety of UME supplementation on lipid metabolism in adults with untreated high LDL-C concentrations. Our study showed that a 500-mg daily supplement of UME administered over 12 weeks positively affected the lipid profiles in adults aged with LDL-C concentrations ranging from 130 to 190 mg/dl. Supplementation of UME over 12 weeks led to a decrease in LDL-C concentration by 17.71 mg/dl, TC concentration by 20.83 mg/dl, and ApoB concentration by 9.22 mg/dl, which was significant compared to the placebo group.

However, it had no favorable effects on the TG, HDL-C, and ApoA concentrations. No AEs were reported in this study. This is consistent with the results of our other previous study (20), and it can be said that the safety of UME has been proven. This may be because the optimal low dose was administered to minimize the possibilities of adverse effects and toxicity but to have lipid-lowering effects (28, 29). Interestingly, diastolic BP decreased at 12 weeks in the UME group compared to the placebo group. A study in spontaneously hypertensive rats reported that prolonged (42 days) administration with UME reduced systolic BP (24). Although it can be assumed that UME has vasorelaxant and antioxidant properties, it is necessary to reconfirm the effect of UME on BP in humans and to conduct further studies on the mechanism.

The mechanism underlying the effects of UME on lipid pathways and metabolism has been reported in a previous animal study (15). Han et al. (15) investigated the impact of UME administration on lipid accumulation in HepG2 cells and hyperlipidemia in HCD-induced Sprague Dawley rats. They observed that, at the treatment concentrations of 50 and 100 μg/ml, UME attenuated OA-induced lipid accumulation *via* activation of the AMPK pathway in a dose-dependent manner. The oral administration of UME decreased the concentrations of TC, TG, and LDL-C and increased the concentration of HDL-C in HCD-induced hyperlipidemia rats. In addition, UME supplementation increased the expression of phosphorylated AMPK and phosphorylated acetyl CoA carboxylase proteins and decreased the expression of the sterol regulatory element binding protein-1 (SREBP-1) and HMGCR proteins in the experimental rats. These results suggest that UME has a favorable ameliorating effect on lipid profiles *via* activation of the AMPK pathway and regulation of lipid metabolism.

Unlike the results of a previous experimental study, which indicated that UME supplementation did not improve all lipid profiles, this human study showed that UME had a positive effect in lowering the TC and LDL-C concentrations but no effect on reducing TG concentration and in raising the HDL-C concentration. Such differences compared to the previous study could be partly explained by a relatively normal range of TG and HDL-C concentrations in both groups at the start of the study. When the TG concentration is higher than 200 mg/dl or the HDL-C concentration is lower than 40 mg/dl, it is traditionally defined as dyslipidemia (30). However, since our study focused on patients with high LDL-C concentrations, TG and HDL-C concentrations were relatively normal at the beginning of the study. Thus, it is presumed that there was no further change when UME supplementation was administered. For this reason, more studies may be needed to verify the effect of UME on lipid profiles in subjects with higher TG or lower HDL-C concentrations.

Epidemiological studies have suggested that ApoB predicts atherosclerotic risk better than traditional TC or LDL-C (31). Among bioactive natural compounds, red yeast rice extract, berberine, and flaxseed have some roles in reducing ApoB concentrations in clinical trials (32). The potential reported mechanisms regarding the effects of nutraceuticals on ApoB are decreased ApoB mRNA expression and secretion, increased upregulation of ApoB receptors, and enhanced protection of ApoB against oxidation (32). In our study, ApoB concentration in the UME group reduced by 9.22 mg/dl (7.8%) compared to that in the placebo group after 12 weeks of treatment. This finding was consistent with that observed in another experimental study (33). In the previous study (33), as in a study investigating the effect of isoflavone on lipid metabolism (34), a decrease in SREBP-2 was also observed. Hwang et al. (33) presumed this as a mechanism of apoB reduction (35), but further studies are warranted to understand the mechanism clearly.

This study has some limitations, including the lack of biological confirmation to determine the mechanism of action of UME on ApoB reduction. Because this study focused on subjects with untreated high LDL-C concentrations (130–190 mg/dl), the effect of UME in patients with elevated TG or low HDL-C concentrations remains unknown. Also, there were hardly any smokers included. The smoking rates for men and women in Korea are 40–50 and 4–8%, respectively (36). Considering that the male-to-female ratio of the subjects of this study was 1:1.8, the overall smoking rate of 5% was very low. Furthermore, physical activity and nutrition intake in this study were assessed by IPAQ and 24-h dietary recall, respectively; therefore, the information may not represent the usual state of participants. Although the lipid-lowering effect of UME decreased more at 12 weeks than at 6 weeks, there is no data for more than 12 weeks, so the impact of using it for more than 12 weeks is unknown. Also, this study did not evaluate whether major adverse cardiovascular events, the endpoint of anti-lipid therapy, could be avoided. Despite these limitations, this study is still considered valuable owing to several strengths. First, to our knowledge, this is the first well-designed clinical study to examine the efficacy and tolerability of UME supplementation in adults with untreated high LDL-C concentrations. Another strength of this study is the use of valid self-report instruments to evaluate participants’ physical activity and dietary intake.

## Conclusion

In conclusion, UME supplementation could improve lipid profiles in adults with high LDL-C concentrations without toxicity or severe adverse effects. However, unlike the results of previous experimental studies, there was no decrease in the concentrations of TG or HDL-C. Further clinical studies are needed to determine the effect of UME supplementation in adults with high TG or low HDL-C concentrations.

## Data availability statement

The original contributions presented in this study are included in the article/Supplementary material, further inquiries can be directed to the corresponding author.

## Ethics statement

The studies involving human participants were reviewed and approved by the Institutional Review Board at Pusan National University Yangsan Hospital. The patients/participants provided their written informed consent to participate in this study.

## Author contributions

SL contributed to the conceptualization of the study, carried out the formal analysis of the data, and coordinated and supervised the entire project. YL and SL designed the methodology of the work, had an active role in the process of participant recruitment and data acquisition, contributed to the validation of results, worked together for data curation, wrote the work’s draft, and reviewed the final document. Both authors contributed to the article and approved the submitted version.

## Footnotes

1
https://clinicaltrials.gov/ct2/show/NCT03773315

2
http://www.r-project.org/
