# Supplementary material for: Potential lipid-lowering effects of Ulmus macrocarpa Hance extract in adults with untreated high low-density lipoprotein cholesterol concentrations: A randomized double-blind placebo-controlled trial
Source: Front Med (Lausanne). 2022 Nov 1;9:1000428. doi: 10.3389/fmed.2022.1000428 (PMC9663492; doi:10.3389/fmed.2022.1000428)
Supplement: Supplementary file 1 [file Data_Sheet_1.PDF]

Potential Lipid-lowering Effects of *Ulmus macrocarpa* Hance Extract in Adults with Untreated High Low-density Lipoprotein Cholesterol Concentrations: A Randomized Double-Blind Placebo-Controlled Trial

Ye Li Lee and Sang Yeoup Lee

SUPPLEMENTARY TABLE 1. Randomization and Batch number.

Registration number	Visits	Batch number	Group
R-001	V2	#NT001-01	Experiment
	V3	#NT001-02	
R-002	V2	#NT002-01	Experiment
	V3	#NT002-02	
R-003	V2	#NT003-01	Control
	V3	#NT003-02	
R-004	V2	#NT004-01	Control
	V3	#NT004-02	
R-005	V2	#NT005-01	Control
	V3	#NT005-02	
R-006	V2	#NT006-01	Experiment
	V3	#NT006-02	
R-007	V2	#NT007-01	Experiment
	V3	#NT007-02	
R-008	V2	#NT008-01	Control
	V3	#NT008-02	
R-009	V2	#NT009-01	Experiment
	V3	#NT009-02	
R-010	V2	#NT010-01	Experiment
	V3	#NT010-02	

R-011	V2	#NT011-01	Control
	V3	#NT011-02	
R-012	V2	#NT012-01	Experiment
	V3	#NT012-02	
R-013	V2	#NT013-01	Experiment
	V3	#NT013-02	
R-014	V2	#NT014-01	Control
	V3	#NT014-02	
R-015	V2	#NT015-01	Experiment
	V3	#NT015-02	
R-016	V2	#NT016-01	Control
	V3	#NT016-02	
R-017	V2	#NT017-01	Control
	V3	#NT017-02	
R-018	V2	#NT018-01	Experiment
	V3	#NT018-02	
R-019	V2	#NT019-01	Control
	V3	#NT009-02	
R-020	V2	#NT020-01	Experiment
	V3	#NT020-02	
R-021	V2	#NT021-01	Control
	V3	#NT021-02	
R-022	V2	#NT022-01	Experiment
	V3	#NT022-02	
R-023	V2	#NT023-01	Control
	V3	#NT023-02	
R-024	V2	#NT024-01	Experiment

	V3	#NT024-02	
R-025	V2	#NT025-01	Control
	V3	#NT025-02	
R-026	V2	#NT026-01	Experiment
	V3	#NT026-02	
R-027	V2	#NT027-01	Control
	V3	#NT027-02	
R-028	V2	#NT028-01	Experiment
	V3	#NT028-02	
R-029	V2	#NT029-01	Control
	V3	#NT029-02	
R-030	V2	#NT030-01	Experiment
	V3	#NT030-02	
R-031	V2	#NT031-01	Control
	V3	#NT031-02	
R-032	V2	#NT032-01	Control
	V3	#NT032-02	
R-033	V2	#NT033-01	Control
	V3	#NT033-02	
R-034	V2	#NT034-01	Experiment
	V3	#NT034-02	
R-035	V2	#NT035-01	Control
	V3	#NT035-02	
R-036	V2	#NT036-01	Experiment
	V3	#NT036-02	
R-037	V2	#NT037-01	Experiment
	V3	#NT037-02	

R-038	V2	#NT038-01	Control
	V3	#NT038-02	
R-039	V2	#NT039-01	Control
	V3	#NT039-02	
R-040	V2	#NT040-01	Experiment
	V3	#NT040-02	
R-041	V2	#NT041-01	Experiment
	V3	#NT041-02	
R-042	V2	#NT042-01	Control
	V3	#NT042-02	
R-043	V2	#NT043-01	Experiment
	V3	#NT043-02	
R-044	V2	#NT044-01	Control
	V3	#NT044-02	
R-045	V2	#NT045-01	Experiment
	V3	#NT045-02	
R-046	V2	#NT046-01	Control
	V3	#NT046-02	
R-047	V2	#NT047-01	Control
	V3	#NT047-02	
R-048	V2	#NT048-01	Experiment
	V3	#NT048-02	
R-049	V2	#NT049-01	Experiment
	V3	#NT049-02	
R-050	V2	#NT050-01	Control
	V3	#NT050-02	
R-051	V2	#NT051-01	Control

	V3	#NT051-02	
R-052	V2	#NT052-01	Control
	V3	#NT052-02	
R-053	V2	#NT053-01	Experiment
	V3	#NT053-02	
R-054	V2	#NT054-01	Control
	V3	#NT054-02	
R-055	V2	#NT055-01	Experiment
	V3	#NT055-02	
R-056	V2	#NT056-01	Experiment
	V3	#NT056-02	
R-057	V2	#NT057-01	Experiment
	V3	#NT057-02	
R-058	V2	#NT058-01	Experiment
	V3	#NT058-02	
R-059	V2	#NT059-01	Control
	V3	#NT059-02	
R-060	V2	#NT060-01	Experiment
	V3	#NT060-02	
R-061	V2	#NT061-01	Control
	V3	#NT061-02	
R-062	V2	#NT062-01	Experiment
	V3	#NT062-02	
R-063	V2	#NT063-01	Experiment
	V3	#NT063-02	
R-064	V2	#NT064-01	Control
	V3	#NT064-02	

R-065	V2	#NT065-01	Control
	V3	#NT065-02	
R-066	V2	#NT066-01	Experiment
	V3	#NT066-02	
R-067	V2	#NT067-01	Control
	V3	#NT067-02	
R-068	V2	#NT068-01	Control
	V3	#NT068-02	
R-069	V2	#NT069-01	Experiment
	V3	#NT069-02	
R-070	V2	#NT070-01	Control
	V3	#NT070-02	
R-071	V2	#NT071-01	Experiment
	V3	#NT071-02	
R-072	V2	#NT072-01	Experiment
	V3	#NT072-02	
R-073	V2	#NT073-01	Experiment
	V3	#NT073-02	
R-074	V2	#NT074-01	Control
	V3	#NT074-02	
R-075	V2	#NT075-01	Control
	V3	#NT075-02	
R-076	V2	#NT076-01	Experiment
	V3	#NT076-02	
R-077	V2	#NT077-01	Control
	V3	#NT077-02	
R-078	V2	#NT078-01	Control

	V3	#NT078-02	
R-079	V2	#NT079-01	Control
	V3	#NT079-02	
R-080	V2	#NT080-01	Experiment
	V3	#NT080-02	
